# Targeting Adipokines in Obesity-Related Tumors

**DOI:** 10.3389/fonc.2021.685923

**Published:** 2021-08-04

**Authors:** Xi Pu, Deyu Chen

**Affiliations:** Institute of Oncology, Affiliated Hospital of Jiangsu University, Zhenjiang, China

**Keywords:** obesity, adipocyte, leptin, insulin, estrogen, adiponectin

## Abstract

Obesity, a global epidemic, is an independent risk factor for the occurrence and development of a variety of tumors, such as breast cancer, pancreatic cancer, ovarian cancer and colorectal cancer. Adipocytes are important endocrine cells in the tumor microenvironment of obesity-related tumors, which can secrete a variety of adipokines (such as leptin, adiponectin, estrogen, resistin, MIF and MCP-1, etc.), among which leptin, adiponectin and estrogen are the most in-depth and valuable ones. These adipokines are closely related to tumorigenesis and the progression of tumors. In recent years, more and more studies have shown that under chronic inflammatory conditions such as obesity, adipocytes secrete more adipokines to promote the tumorigenesis and development of tumors. However, it is worth noting that although adiponectin is also secreted by adipocytes, it has an anti-tumor effect, and can cross-talk with other adipokines (such as leptin and estrogen) and insulin to play an anti-tumor effect together. In addition, obesity is the main cause of insulin resistance, which can lead to the increase of the expression levels of insulin and insulin-like growth factor (IGF). As important regulators of blood glucose and lipid metabolism, insulin and IGF also play an important role in the progress of obesity related tumors. In view of the important role of adipokines secreted by adipocytes and insulin/IGF in tumors, this article not only elaborates leptin, adiponectin and estrogen secreted by adipocytes and their mechanism of action in the development of obesity- related tumors, but also introduces the relationship between insulin/IGF, a regulator of lipid metabolism, and obesity related tumors. At the same time, it briefly describes the cancer-promoting mechanism of resistin, MIF and MCP-1 in obesity-related tumors, and finally summarizes the specific treatment opinions and measures for various adipokines and insulin/insulin-like growth factors in recent years.

## Introduction

**Figure 1:**
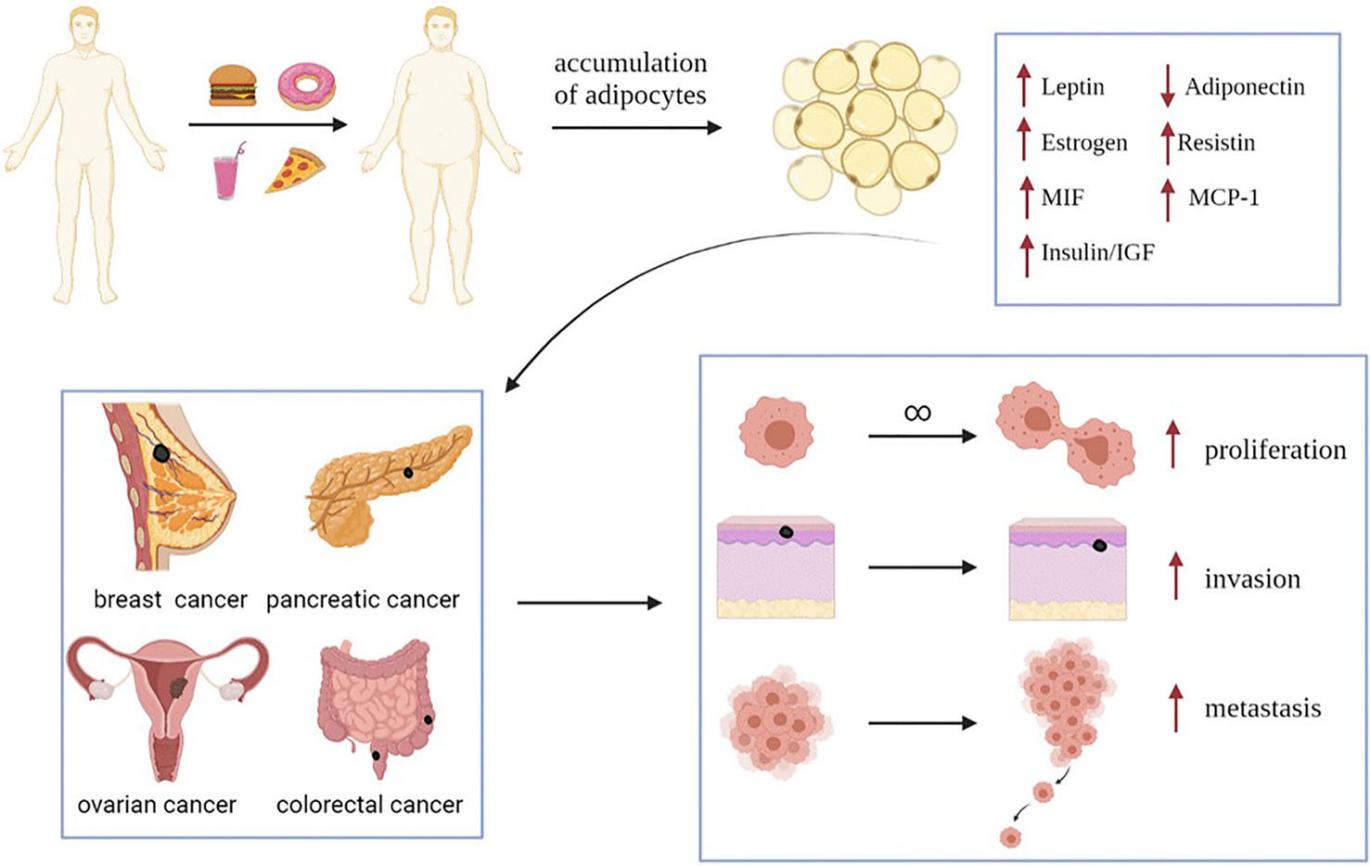
Obesity increased the expression of leptin, estrogen, resistin, MIF, MCP-1 and insulin/IGF, and decreased the expression of adiponectin. These changes in the expression levels of adipokines and hormones will promote the progress of obesity related-tumors (such as breast cancer, pancreatic cancer, ovarian cancer and colorectal cancer), and promote the proliferation, invasion and metastasis of tumor cells. MIF, Macrophage migration inhibitory factor; MCP-1, Monocyte chemoattractant protein-1; IGF, insulin-like growth factor.

Obesity is an increasingly serious problem in society. According to a statistical data from 1975 to 2014, the number of obese people in the world increased from 150 million in 1975 to 641 million in 2014, and the number of obese people in China ranks first in the world (1). According to the current trend, the global prevalence of obesity will continue to rise from 2019 to 2024 (1). Obesity is a key problem associated with human survival and development that cannot be ignored (1). As an independent risk factor for many types of tumors, obesity also plays important roles in the occurrence and development of tumors. The chronic inflammatory process of obesity is also beneficial to the development of tumors. Obesity can increase the risk of breast cancer in postmenopausal women (2), affect the prognosis of pancreatic cancer (3), promote ovarian cancer cells to the greater omentum (mainly composed of adipocytes) (4), and increase the postoperative mortality of colorectal cancer patients (**Figure 1**) (5).

Adipose tissue is an endocrine organ, and adipocytes are an important part of adipose tissue and play an important role in the tumor microenvironment. Adipocytes can be divided into white adipocytes and brown adipocytes (6). Leptin is mainly secreted by white fat cells and plays a key role in regulating energy balance and maintaining metabolic stability (7). In recent years, a large number of studies have shown that leptin affects the occurrence and development of many obesity related tumors. However, adiponectin, also secreted by adipocytes, plays a significant role in cancer suppression because of its anti-tumor effect and is known as “Guardian Angel Adipocyte Factor”. High expression of leptin and low levels of adiponectin in obese patients have become the prerequisites for the occurrence of obesity-related tumors.

Obese people also produce high levels of tumor-promoting hormone, estrogen. In addition, estrogen can promote the deposition and function of adipose tissue directly or by activating their receptors in adipocytes and adipose tissue (8), which suggests that estrogen has a two-way regulatory effect on adipose tissue. In general, excessive estrogen levels will promote tumor progression, but estrogen deficiency can also lead to imbalances in glucose metabolism and lipid metabolism. Estrogen binds to estrogen receptor-α (ER-α) to play a cancer-promoting effect in breast and ovarian cancer. In breast cancer, about 70% of breast tumors express estrogen receptor α which is called ER positive breast cancer and relies on endogenous estrogen to promote the proliferation and metastasis of tumor cells (9). Interestingly, the two receptors of estrogen, ER-α and ER-β, play completely opposite roles, which also provides different ideas for the treatment of tumors.

Insulin resistance is common in obese patients. A variety of adipokines, such as leptin and MIF, are related to the occurrence of insulin resistance. The higher the degree of insulin resistance, the more sensitive the pancreatic β-cells are to the increase in blood glucose concentration, which makes the pancreatic β-cells secrete more insulin to lower blood sugar (10). Therefore, a large number of studies have combined insulin/insulin like growth factor with obesity-related tumors.

Furthermore, the progression of obesity-related tumors is determined by the interaction of many factors, and resistin, MIF and MCP-1 play an important role in this process.

In recent years, a large number of studies have explored the relationship between obesity and tumorigenesis and tumor development, especially the mechanism of adipokines secreted by adipocytes in tumor microenvironment. Therefore, this paper not only describes the effects of leptin, adiponectin and estrogen on obesity related tumors in detail, but also specifically points out the mechanism of action of insulin and insulin-like growth factors that are commonly associated with obesity, insulin resistance and obesity-related tumors. The interaction of adiponectin with leptin, insulin and estrogen is also discussed, which provides a promising therapeutic target for the treatment of obesity related tumors. In addition, this paper also briefly describes the mechanism of action of the other three adipokines, resistin, MIF and MCP-1 in tumor and their therapeutic strategies.

## Leptin

**Figure 2:**
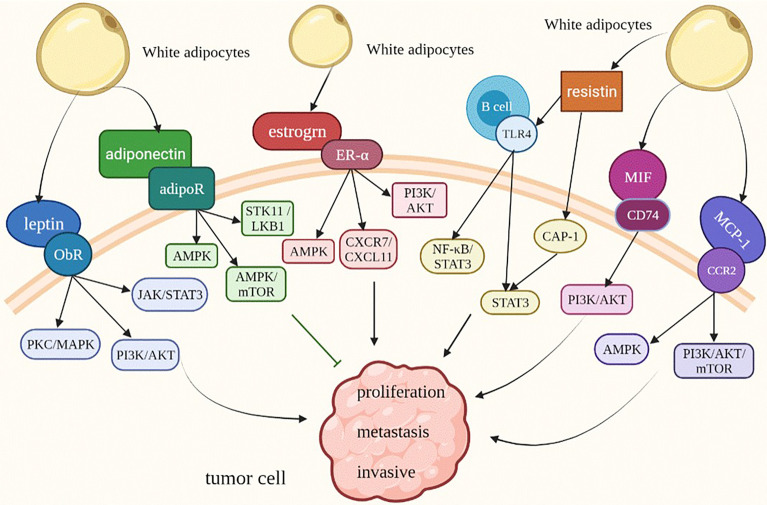
Adipokines modify the behavior of obesity-related tumor cells. ObR, leptin receptor; JAK/STAT3, Janus kinase/signal transducer and activator of transcription 3; MAPK/ERK, mitogen-activated protein kinase/extractor signal regulated kinase; PI3K/AKT, phosphoinositide-3 kinase/protein kinase B; AMPK, AMP-activated protein kinase; STK11/LKB1, Serine/threonine kinase 11/Liver Kinase B1; TLR4, Toll-like receptor 4; CAP1, adenylate cyclase-related protein 1.

Leptin, which is a 16 kDa hormone mainly synthesized and secreted by white adipocytes (11), acts as a regulating factor of food intake through the role of hypothalamus (12). In 1994, researchers successfully cloned the mouse OB gene and human homologous sequence by using location-based cloning technology for the first time, and predicted OB protein by its gene, which laid a solid foundation for later research on leptin (13). In the past 20 years, leptin has mainly been used in research and treatment of leptin deficiency-induced obesity and typical obesity (14). After leptin transmits obesity signals to the brain, it can maintain energy balance and regulate weight by regulating the activities of neurons in multiple areas of the hypothalamus (15). Research on the long subtype of leptin receptor, which is the main receptor, is the most significant. However, a large number of studies have found that abnormal binding of leptin and its receptor (ObR) can promote the occurrence, development, metastasis and invasion of some tumors. Especially in obese patients, the plasma leptin concentration is directly proportional to the fat quality. High expression of leptin and an increase in circulating leptin levels further promote the progression of many cancers, such as breast cancer, pancreatic duct cancer and ovarian cancer. Moreover, leptin can lead to lipid accumulation and further aggravate obesity (16). Therefore, for obese patients, the overexpression of leptin and leptin receptors is the key to the development of various obesity-related tumors. Leptin receptors include the long type and short type. Leptin binds to leptin receptors to activate the Janus kinase/signal transducer and activator of transcription (JAK/STAT), mitogen-activated protein kinase/extractor signal regulated kinase (MAPK/ERK) and phosphoinositide-3 kinase/protein kinase B (PI3K/AKT) (17–19), and the expression of multiple genes in these signaling pathways promotes the proliferation and metastasis of tumor cells (**Figure 2**). Thus, leptin should be regarded as an important target in the treatment of some tumors.

### Leptin and Tumorigenesis

Leptin produced in adipose tissue can directly affect tumor development through its receptor (ObR) (20). Leptin is an important mediator to linking obesity and a variety of obesity related tumors. Leptin can increase the risk of obesity related cancer, especially hormone dependent tumors, such as breast cancer, endometrial cancer and ovarian cancer. Leptin has been found to accelerate the occurrence and development of breast cancer, and obese women are more likely to suffer from breast cancer with high malignancy (21). Obesity has a more serious impact on postmenopausal women than premenopausal women suffering from breast cancer (13, 18), which is caused by the different levels of estrogen secretion, and leptin plays a key role in this process. Leptin increases the risk of breast cancer by increasing the effect of hormone secretion on breast tissue hyperplasia, which is more significant in postmenopausal women (22). Before menopause, the body maintains a balance of estrogen levels through the negative feedback regulation mechanism of the hypothalamus-pituitary-gonad axis. However, in postmenopausal women, although the ovarian function deteriorates, an increase in adipose-derived leptin will not only induce the production of estrogen mediated by aromatase in MCF7 breast cancer cells (23), but also activate the signal transduction of estrogen receptor (ER) (23, 24), which will increase the level of estrogen. In addition, aromatase is highly expressed in breast cancer, which indicates that the development of breast cancer may be closely related to aromatase. Aromatase, which is the rate-limiting enzyme in estrogen biosynthesis, plays an important role in promoting the production of estrogen. It has also been found that leptin induces the expression of aromatase genes by affecting PKC/MAPK signaling (**Table 1**), which ultimately leads to an increase in the estrogen expression level, increasing the risk of breast cancer and promoting the development of breast cancer (25). Leptin is the signal amplifier of estrogen in tumor epithelial cells. It can promote tumor progression by activating the signaling pathways, not only in breast cancer, but also in other obesity-related tumors, such as ovarian cancer and endometrial cancer. Ovaries are the main source of estrogen, and estrogen can promote the development of epithelial ovarian cancer, ERα plays a key role in epithelial ovarian cancer and can participate in leptin induced cell invasion by increasing the expression of MMP9 (50). ERα instead of ERβ can participate in the invasion of ovarian cancer cells induced by leptin in an estrogen-independent manner (50), and 17β-estradiol can reverse the migration of ovarian cancer cells induced by leptin through the PI3K/Akt signaling pathway (32). Therefore, in ovarian cancer, the effect of estrogen and leptin on tumor is more complex. The exact molecular mechanism of the reaction between estrogen and leptin needs to be further explored. The increased risk of endometrial cancer in obese women suggests that leptin secreted by adipose tissue affects the development of endometrial cancer (51). At the same time, the expression of leptin receptor is also regulated by ER and progesterone receptor (PR), but the specific mechanism is unclear (20). In conclusion, leptin secreted by adipose tissue can stimulate overexpression of estrogen and its receptor through various signaling pathways, which can increase the risk of multiple cancers and provide new ideas for the prevention of such tumors.

**Table 1:** Effect of Adipokines on Tumor Progression and its Mechanism.

Adipokines	Receptor	Secretory Cell	Tumor Type	Cancer Development	Mechanism	Reference
Leptin	ObR	White adipocytes	Breast cancer	↑	PKC/ERK1/2, PI3K/AKT, JAK/AKT/STAT	(25–29)
			Pancreatic cancer	↑	JAK2/STAT3, PI3K/AKT	(30, 31)
			Ovarian cancer	↑	PI3K/AKT, MEK/ERK1/2	(32–34)
Adiponectin	AdipoR	White adipocytes	Breast cancer	↓	AMPK,STK11/LKB1	(35, 36)
			Pancreatic cancer	↓	Insulin sensitizer	(37)
			Colorectal cancer	↓	AMPK/mTOR	(38)
Estrogen	ERα	White adipocytes	Breast cancer	↑	PI3K/AKT, MAPK	(39)
			Ovarian cancer	↑	CXCR7/CXCL11	(40)
	ERβ		Breast cancer	↓	PI3K/AKT	(41)
			Ovarian cancer	↓		(42, 43)
Resistin	CAP-1/TLR4	White adipocytes	Breast cancer	↑	NF-κB/STAT3	(44)
			Pancreatic cancer	↑	STAT3	(45)
MIF	CD74	White adipocytes	Breast cancer	↑	PI3K/AKT	(46)
			Pancreatic cancer	↑	MIF-miR-301b-NR3C2	(47)
MCP-1	CCR2	White adipocytes	Breast cancer	↑	AMPK	(48)
			Ovarian cancer	↑	PI3K/AKT/mTOR	(49)

↑ represents promote, ↓ represent suppress.

### Leptin and Progression of Obesity-Related Tumors

Epithelial mesenchymal transition (EMT) plays an important role in the development of tumor by enhancing invasion ability, which worsen the prognosis of tumors, and leptin plays a key role in this process. Although the molecular mechanism is not clear, it is necessary to induce the activation of different signaling pathways. The overexpression of leptin in obese patients can promote the development of obesity related triple negative breast cancer (TNBC) by promoting the accumulation of tumors stem cells (CSCs) and EMT (52). Leptin can upregulate the expression of pyruvate kinase M2 (PKM2) (one of the main rate-limiting enzymes in glycolysis that is over expression in a variety of tumors). Leptin can promote EMT in breast cancer cells by upregulating the expression of PKM2 and activating the PI3K/AKT signaling pathway, while antagonizing leptin receptors and the PI3K/AKT signaling pathway can block the EMT phenomenon induced by leptin, which provides a new idea for the treatment of breast cancer (26). In addition to promoting cancer cell metastasis through EMT, leptin can also promote the expression of lysine hydroxylase-2 (PLOD2) by activating the PI3K/AKT signaling pathway, thus promoting the metastasis of breast cancer (27). It can also upregulate the expression of sterol regulatory element binding protein-1 (SREBP1) through the PI3K/AKT signaling pathway, thus promoting the expression of cholesterol acetyltransferase-2 (ACAT2), activating ObR and promoting breast cancer metastasis, proliferation and migration and invasion of breast cancer cells (28). Aiming at the protective barrier CNN5 of the breast, as a cytokine that can inhibit tumor growth and invasion, leptin can continuously inhibit the protective effect of CCN5 by activating the JAK/AKT/STAT signaling pathway, and promote the growth and progression of breast cancer (29).

Leptin also promotes the development of a variety of obesity related tumors, such as pancreatic cancer, ovarian cancer and colon cancer. Leptin can specifically bind to the leptin receptor (ObR) expressed in pancreatic cancer and promote the migration and invasion of pancreatic cancer cells but does not affect the proliferation ability of pancreatic cancer cells (30). Therefore, increasing the expression of leptin receptors can help the growth and development of pancreatic cancer. Leptin upregulates the expression of matrix metalloproteinase-13 (MMP-13) through the JAK2/STAT3 signaling pathway and enhances the invasion ability of pancreatic cancer cells (31). Leptin can also promote the migration of pancreatic cancer cells by over activating the PI3K/AKT signaling pathway, thus promoting the growth of pancreatic cancer (30). Ovarian cancer (OVCA) is also an obesity related cancer. Accumulating studies have shown that leptin has a certain impact on the growth of ovarian cancer, which provides a direct link between obesity and the progression of ovarian cancer. Leptin binds to leptin receptors, activates the MEK/ERK1/2 and PI3K/Akt signaling pathways (33, 34), upregulates the expression of cyclin D1 and anti-apoptotic factor Mcl-1, induces the proliferation and growth of ovarian cancer cells, inhibits apoptosis, and worsens the prognosis of patients with ovarian cancer. High expression of leptin and leptin receptors in colon cancer provides a precondition for the development of colon cancer (53).

## Adiponectin

Adiponectin, a serum protein with a molecular weight of 30 kDa that is regulated by insulin, was discovered in 1995. Because of its molecular weight, it was named adipocyte complement-associated protein 30 kDa (Acrp30) (54). Adiponectin is also a cytokine secreted by adipose tissue and a protein secreted by mature adipocytes and is closely related to adipose tissue and obesity (**Figure 2**) (55). The expression level of adiponectin is connected with sex, and the expression level of adiponectin in women is twice than that in men (56).

### Anti-Tumor Effect of Adiponectin

Although most of the adipokines secreted by adipose tissue are positively correlated with tumor progression, adiponectin has attracted scientists’ attention for its unique anti-tumor effect and thus is also called “Guardian Angel adipocytokine”. Adiponectin can not only inhibit tumor progression by regulating tumor cell proliferation and inducing the apoptosis response but also has unique anti-inflammatory and anti-insulin resistance effects that are very important for the treatment of obesity induced tumors, especially in breast cancer, pancreatic cancer and colorectal cancer (57). It is expected to become a therapeutic target for a variety of obesity related tumors.

Studies have found that low plasma adiponectin levels will increase the risk of breast cancer in premenopausal and postmenopausal women, especially in luminal breast cancer and postmenopausal HER-2-enriched breast cancer, which shows that adiponectin has an anti-breast cancer effect (58, 59). The negative effect of adiponectin on cell growth is mainly due to the increase in cell number in G1/G0 phase and the induction of apoptosis. Long-term exposure to adiponectin can reduce the expression of cyclin D1 and E2 and inhibit cell proliferation (60). Adiponectin can induce AMPK activation through serine/threonine kinase 11/Liver Kinase B1 (STK11/LKB1) (**Table 1**). As a tumor suppressor, AMP-activated protein kinase (AMPK) can inhibit cancer cell proliferation and promote cancer cell apoptosis (35). AMPK activation promotes the expression and phosphorylation of serine/threonine kinase ulk1, leading to the occurrence of cytotoxic autophagy in breast cancer cells and inhibiting tumor growth (36). The regulation of AMPK activity provides a new strategy for the treatment of breast cancer.

Adiponectin is not only an insulin sensitizer, but also has an anti-inflammatory effect (37). It also has a prominent effect on the progression of pancreatic cancer. According to clinical studies, low circulating adiponectin levels before diagnosis are associated with increased risk of pancreatic cancer (61). In pancreatic cancer, when the adiponectin receptor gene is knocked out, the antiproliferative effect of adiponectin can be completely eliminated. Therefore, adiponectin can also inhibit the progression of pancreatic cancer by inhibiting pancreatic cancer cells proliferation and inducing apoptosis. Studies have found that adiponectin can inhibit the accumulation of β - Catenin in pancreatic cancer cells by inactivating GSK-3β, thereby reducing the expression of cyclin D1 and leading to arrest of the pancreatic cancer cell cycle in G-G phase, which plays a protective role in pancreatic cancer (62). Therefore, the expression level of adiponectin is closely related to the development of pancreatic cancer. Increasing the expression level of adiponectin in patients with pancreatic cancer may be a key measure to slow down the progression of pancreatic cancer and improve the prognosis of pancreatic cancer.

Although there are few studies on the mechanism by which adiponectin regulates colorectal cancer cells, it is certain that adiponectin plays an anti-tumor role in colorectal cancer. Adiponectin mainly acts through adiponectin receptors (AdipoR1 and AdipoR2). AdipoR1 and AdipoR2 were found to be expressed in 72% and 68% of colorectal cancer tissues, respectively. In general, adiponectin receptor (AdipoR) expression was strongly positive in normal epithelium but weakly positive or even negative in colorectal cancer tissues (63). A high level of adiponectin circulation in the body is associated with a lower risk of colorectal cancer and a higher mortality rate, which is reflected in the negative correlation between the expression level of adiponectin and the tumor grade of colorectal cancer (64–66). High adiponectin levels can also inhibit the progression of colorectal cancer by interfering with the cancer-promoting effect of leptin (67). Studies have found that adiponectin can inhibit the proliferation of colon cancer cells induced by interleukin-6 (IL-6) by inhibiting the activation and phosphorylation of STAT3 and thus has an anti-tumor effect (68). In colorectal cancer, adiponectin can also inhibit the growth of cancer cells by activating the tumor suppressor AMPK and downregulating the target of rapamycin (mTOR) pathway (38).

## Estrogen

Estrogen refers to a group of hormones (such as 17 β - estradiol, estrone and estriol) that are primarily secreted by the ovary of female animals and have the function of promoting the development of secondary sexual characteristics and the maturation of sexual organs in female animals (69). Estrogen acts through three different receptors: estrogen receptor α (ER α), estrogen receptor β (ER β) and G protein-coupled estrogen receptor (GPER). The expression of estrogen receptor increases with age (70). The production and regulation of estrogen *in vivo* cannot be separated from the hypothalamus and pituitary. The hypothalamus stimulates the pituitary gland to secrete gonadotropin (GN) by releasing gonadotropin releasing hormone (GnRH). Gonadotropins include luteinizing hormone (LH) and follicle stimulating hormone (FSH), which play a synergistic role in stimulating the production and secretion of ovarian sex hormones (71). In addition to the ovary, estrogen can also be secreted by adipocytes, especially in postmenopausal women. Adipose tissue, as an endocrine organ, becomes the main site of estrogen production (72, 73). Therefore, estrogen, as an adipokine, plays an important role in obesity related tumors (**Figure 2**).

In obese people, the increase in estrogen level is related to two factors: on the one hand, with the excessive accumulation of adipose tissue, the level of estrogen secreted will also increase; on the other hand, it is closely related to white adipose tissue (WAT) and inflammation. White adipose tissue is one of the adipose tissue types (the other two main types are beige adipose tissue and brown adipose tissue), and WAT has the ability to secrete estrogen (69). Its ability is more prominent in obese people, because obesity is closely related to chronic fatty inflammation. In the process of obesity progression, adipocytes can recruit and activate macrophages through CCL2/IL-1β/CXCL12 signaling pathway (**Table 1**) (21). Macrophages surround dead or dying adipocytes to form tree crown structures (CLSs), which are white adipose tissue inflammation (WATi), another characteristic of obesity (74). Inflammation of white adipose tissue is related to the increase in the aromatase gene expression level and estrogen secretion, which can stimulate the expression of aromatase. Then aromatase converts androgen in adipose tissue into estrogen, which increases estrogen expression level (69, 72, 75). Aromatase is a rate limiting enzyme in estrogen biosynthesis. Its expression in adipose stromal cells of the breast is believed to promote the growth of breast tumors (71), and promote the progression of ovarian cancer. The relationship among obesity, white adipose tissue inflammation, aromatase and estrogen expression levels is complex, and the mechanism is not clear, but this does not hinder us from analyzing and observing the association of these four factors with the occurrence and development of tumors. Since the ovary is the main source of estrogen before menopause, and adipose tissue is the main source of estrogen after menopause, we mainly discuss the key role of estrogen and its receptor in the pathogenesis of breast cancer (rich in adipose tissue) and ovarian cancer (the main source of premenopausal estrogen).

### Estrogen and Breast Cancer

The progression of breast cancer is closely related to estrogen levels. Most breast cancer is estrogen dependent (76). Clinical studies have found that the level of estrogen and its metabolites in postmenopausal women is related to the incidence rate of breast cancer (77). In addition, the absence of UDP-glucuronosyltransferases (UGTs), which are involved in the estrogen clearance process, can not only cause estrogen accumulation, but also promote the progression of breast cancer, indirectly indicating that estrogen has a promoting effect on breast cancer (78). Although the ovary is the main source of estrogen in premenopausal women, most breast cancer is diagnosed after menopause (79). As mentioned above, estrogen in postmenopausal women is mainly catalyzed by its precursor androgen under the mediation of aromatase. Obesity can increase the expression of aromatase by triggering a series of inflammatory reactions, thus promoting an increase in estrogen levels. Therefore, obesity is a risk factor for postmenopausal women with breast cancer (which also has an impact on premenopausal breast cancer patients), and estrogen will further increase the risk of breast cancer in postmenopausal women. Therefore, whether a patient is premenopausal or postmenopausal, estrogen has a significant impact on breast cancer.

The effect of obesity on breast cancer is reflected not only in its ability to increase the expression level of estrogen but also in its effect on estrogen receptors, especially in postmenopausal women with positive for estrogen receptor α (ER α) (80, 81). Based on the collected serum of postmenopausal breast cancer patients, it was found that circulating factors in the serum of postmenopausal obese women can enhance the cross-talk between the IGF-IR, PI3K/AKT and MAPK signaling pathways with nongenomic ERα signaling, which stimulates the survival and growth of Erα-positive breast cancer cells, increases the resistance to aromatase inhibitor therapy and impacts the therapeutic effect (39). It further shows that obesity may promote the progression of ERα-positive postmenopausal breast cancer. The treatment of such tumors should pay attention to the control of estrogen.

It is worth noting that, unlike the well-known tumor-promoting ability of ERα, the effect of estrogen receptor β (ERβ) on tumors is not completely clear (82), and some studies have even suggested that ERβ is a tumor suppressor (42). An increase in its expression level helps to improve the effect of tumor treatment and improve the survival rate and can be used as a potential target for tumor therapy (83–85). The tumor-promoting effect of obesity may be related to a decrease in ERβ expression. Systemic factors related to obesity, such as interleukin-6 receptor (IL-6R), leptin receptor (OB-R) and insulin-like growth factor-I receptor (IGF-IR), can inhibit the expression of ERβ in breast cancer cells through the pathway mediated by human epidermal growth factor receptor 2 (HER2) so that cancer cells can obtain greater cell activity, thus promoting their growth and worsening the prognosis of breast cancer (83). ERβ is expressed in a large proportion of triple-negative breast cancer (TNBC) that does not express Erα (76). Although the role of ERβ in TNBC is still unclear, a considerable number of studies have suggested that ERβ expression is beneficial to the treatment and prognosis of triple negative breast cancer, because ERβ can indirectly reduce the activation of androgen receptor (AR) by inhibiting the PI3K/AKT signaling pathway, and plays an anti-cancer role in AR+ TNBC (41). On the flip side, ERβ can inhibit the proliferation and invasion of tumor cells (85) and inhibit the signal transduction of transforming growth factor beta by mediating cysteine protease inhibitors to inhibit TNBC metastasis (86). So ERβ agonists may be a good choice for the treatment of this type of tumor.

### Estrogen and Ovarian Cancer

The ovary is the main source of estrogen in premenopausal women. Although the results are not always consistent (87), in general, estrogen not only increases the risk of ovarian cancer (88, 89), but promotes tumor progression through its mitogenic ability in the early stage of ovarian cancer (90). Most ovarian cancer patients have no symptoms before diagnosis (91), and more than 70% of ovarian cancers are stage III at the time of diagnosis (92), which is one of the reasons for the high mortality of ovarian cancer. Therefore, it is very important to find early detection markers of ovarian cancer to achieve early diagnosis and early treatment and reduce mortality. Estrogen metabolic imbalance may be associated with the risk of ovarian cancer (87). The reaction between its metabolites and DNA leads to formation estrogen DNA adducts, which are not only a key factor in the occurrence of ovarian cancer but can also be used as an early diagnostic marker for the risk of ovarian cancer (92). Estrogen DNA adducts are crucial for early diagnosis of ovarian cancer and improving the cancer survival rate.

It is well known that epithelial mesenchymal transition (EMT) plays an active role in tumor metastasis. In this process, epithelial cells with adhesion functions transform into interstitial cells, which allows the cells to metastasize and invade. Loss of E-cadherin expression is considered to be a key step in carcinogenesis and EMT. It has been found that 17 β-estradiol (E2) can not only inhibit the activity and expression of E-cadherin but also activate the promoter activity of snail and slug, which are related to EMT, and promote upregulation of expression to induce the migration of cancer cells and promote ovarian cancer progression (93). Estrogen can also play a role in EMT through estrogen receptor α (ERα). In ERα-positive ovarian cancer, estrogen can not only induce activation of the CXCR7/CXCL11 axis through recruitment of ERα, thus increasing the expression of CXCR7, an estrogen-responsive gene, it can enhance the expression of chemokine ligand I-TAC/CXCL11, which leads to phosphorylation of the CXCR7 promoter site ser-118 and the recruitment of the estrogen receptor ERα, thus realizing positive feedback regulation (40). The synergistic effect of estrogen, ERα and the CXCR7 signaling axis promotes the EMT pathway and induces the invasion phenotype of ovarian cancer cells.

Similar to breast cancer, ERβ also antagonizes the metastasis promoting effect of ERα in ovarian cancer (93, 94). ERα is generally considered to promote the proliferation, migration and progression of cancer cells (95). In one clinical study, 78% of ovarian cancers were ERα positive (96). However, the opposite function of ERβ can inhibit the growth and invasion of cancer cells and promote apoptosis (42, 43). Therefore, the overall survival rate of ovarian cancer patients with strong ERβ expression is significantly higher than that of ovarian cancer patients with weak ERβ expression (97). In recent years, these studies have explored the mechanism of ERβ in ovarian cancer. Precisely because these two estrogen receptors have opposite effects, the imbalance in ERβ and ERα expression (low ERβ expression and high ERα expression) may be a preconditions for the occurrence and development of tumors and provide a new therapeutic target for ovarian cancer and other hormone-dependent tumors (such as breast cancer) (94). Here we focus on the role of ERβ in ovarian cancer identified in recent years.

Low ERβ expression is a common feature of *in situ* ovarian epithelial tumors. With decreasing ERβ expression levels, the proliferation and migration ability of cancer cells gradually improve. However, in metastatic tumors, only ERα was found to be expressed, and ERβ was completely absent (98). This further indicates that the imbalance in ERβ and ERα expression not only affects the risk of tumor occurrence but also promotes tumor progression and even affects tumor prognosis.

## Other Adipokines

### Resistin

The resistin secreted by adipose tissue is also closely related to tumor progression, and it is also an important bridge connecting obesity and insulin resistance (**Figure 2**). Like leptin, resistin increases the risk of breast cancer. Serum resistin levels in breast cancer patients were positively correlated with tumor size, malignancy and lymph node metastasis and decreased disease-free and overall survival rates were observed in the high resistin expressing patients (99). These characteristics also exist in pancreatic cancer ovarian cancer (100, 101).

The effect of resistin on tumors is mainly reflected in epithelial mesenchymal transition.

Resistin mediates breast cancer epithelial-mesenchymal transition through adenylate cyclase-related protein 1 (CAP1), thereby promoting breast cancer cell metastasis (**Table 1**) (102). What’s more, resistin activates the Toll-like receptor 4 (TLR4) of B cells and mediates the NF-κB/STAT3 signaling pathway, which can also promote epithelial-mesenchymal transition (44). Resistin signaling depends on the binding of resistin to its receptor, and both CAP1 and TLR4 are receptors for resistin. Resistin can also promote the progression of pancreatic cancer by binding to these two receptors. Resistin combines with CAP1 and TLR4 to promote the proliferation, migration and invasion of pancreatic cancer cells through the STAT3 signaling pathway (45). Similarly, resistin also plays a role in promoting epithelial-mesenchymal transition in ovarian cancer. Although the mechanism is unclear, studies have found that resistin can down-regulate E-cadherin and up-regulate ZEB1 and vimentin (101).

In conclusion, resistin is closely related to tumorigenesis and tumor development, and its cancer-promoting effect is mainly manifested in the induction of epithelial mesenchymal transition. Although the mechanism of action of resistin is still unclear, it does not prevent us from using it as a target for tumor therapy.

### Macrophage Migration Inhibitory Factor (MIF)

Obesity is considered to be a chronic inflammatory process, which can promote inflammation in adipose tissue. Macrophage migration inhibitory factor (MIF), an immunomodulatory cytokine, is considered a key mediator of inflammatory response. It is released by human pre-adipocytes and mature adipocytes, and the amount of release is proportional to body mass index (103). In addition, MIF is a positive regulator of insulin, which can stimulate islets β cells secrete insulin and participate in glucose metabolism (104). This shows that the higher BMI, the more serious the accumulation of adipocytes, the more MIF and insulin secretion, the more serious the impact on occurrence of insulin resistance and tumor progression (**Figure 2**).

Due to the increase in body fat of obese patients, the secretion of MIF increases. MIF overexpression is frequently observed in a variety of human cancer types, including breast cancer, pancreatic cancer and ovarian cancer. High expression of MIF is associated with tumor aggressiveness and poor patient outcomes. Overexpression of MIF in breast cancer is closely related to tumor growth and metastasis. MIF can effectively inhibit breast cancer cell apoptosis by activating PI3K/AKT pathway (**Table 1**) (46). However, the absence of MIF can increase the expression of CD4^+^T and CD8^+^T in tumor tissue, activate more dendritic cells, promote the occurrence of immunogenic cell death, and produce severe anti-tumor immune response (105), which provides a direction for the treatment of tumors with high expression of MIF.

MIF also serves important roles in pancreatic ductal adenocarcinoma (PDAC). MIF is an independent risk factor for poor survival of pancreatic cancer, which can promote the activation of AKT and extracellular signal-regulated kinases, up-regulate the expression of cyclin D1 and matrix metalloproteinase 2 (MMP-2), thereby promoting the invasion and metastasis of pancreatic cancer cells (106). Nuclear receptor subfamily 3, group C, member 2 (NR3C2), a tumor suppressor, has a positive correlation with survival rate and can inhibit epithelial mesenchymal transition. However, in PDAC with high MIF expression, the expression level of nr3c2 was relatively low (47), which indirectly proved that MIF was positively correlated with the mortality and invasion ability of pancreatic cancer cells.

MIF is also highly expressed in patients with ovarian cancer, which is related to the development of ovarian cancer. However, in recent years, more studies have focused on the relationship between MIF and early diagnosis of ovarian cancer. Early detection of ovarian cancer can reduce mortality, MIF plays an important role in early detection of ovarian cancer. MIF, as one of the biomarkers for early detection of ovarian cancer, combined with CA125, OPN and anti-IL-8 autoantibody can increase the detection rate of early ovarian cancer to 82%, which significantly improves the detection rate of early ovarian cancer (107).

In general, MIF may be a potential therapeutic target for obesity related tumors and it also has a significant contribution to improve the early detection rate of ovarian cancer.

### Monocyte Chemoattractant Protein-1 (MCP-1)

Monocyte chemoattractant protein-1 (MCP-1), secreted by adipocytes, is another key factor of macrophage infiltration in adipose tissue of obese patients. Furthermore, MCP-1 is also an adipokine associated with the development and progression of cancer (**Figure 2**).

Compared with non-obese breast cancer patients, the expression level of MCP-1 in the serum of obese breast cancer patients is higher. MCP-1 induces EMT and tumor cell invasion (especially triple-negative breast cancer) by binding to its receptor cysteine-cysteine chemokine receptor 2 (CCR2), promoting p44/p42 phosphorylation and inducing AMPK signal pathway, or mediate RK/GSK-3β/Snail signal pathway (**Table 1**) (48, 108). In addition to promoting the invasion of breast cancer cells, MCP-1 plays an important role in the tumor microenvironment by recruiting macrophages, allowing a large number of macrophages to infiltrate in the tumor microenvironment (109). These macrophages are known as tumor-associated macrophages (TAM). One of the characteristics of inflammatory breast cancer is the high infiltration of TMA which can act on tumor cells by secreting a variety of cytokines to promote the growth, proliferation, and metastasis of tumor cells. Therefore, MCP-1 may be a potential therapeutic target for inflammatory breast cancer and triple negative breast cancer. It should be noted that when chemotherapeutic drugs (such as paclitaxel or carboplatin) are used to treat tumors, the role of MCP-1 in promoting tumor cell metastasis is also worthy of our attention. This is because the use of chemotherapeutic drugs alone or in combination will lead to an increase in the expression level of MCP-1 (110), which may lead to the failure of tumor treatment and even worsen the condition. Therefore, the use of chemotherapy drugs in the treatment of breast cancer at the same time need to cooperate with anti-MCP-1 treatment is expected to achieve the desired therapeutic effect.

MCP-1 can also regulate the progression of pancreatic cancer, but recent studies have focused on MCP-1 as a marker of pancreatic cancer. MCP-1 can be used as a biomarker of pancreatic cancer. In pancreatic cancer, MCP-1 expression is increased in untreated cachexia patients compared with patients with stable body weight, so it can be used as a potential biomarker of cancer cachexia (111).

Ovarian cancer cells tend to migrate to omentum rich in adipocytes, and MCP-1 produced by omental adipocytes plays a key role in this process. MCP-1 specifically binds to CCR2 and activates PI3K/AKT/mTOR signaling pathway and its downstream factor hypoxia inducible factor-1α (HIF-1α) and vascular endothelial growth factor -A (VEGF-A), facilitating ovarian cancer cell metastasis to omentum (49).

In summary, the cancer-promoting effect of MCP-1 is inseparable from binding to its cognate receptor CCR2. Therefore, therapeutic strategies targeting the MCP-1/CCR2 pathway may be a promising therapeutic target to prevent the progression of obesity-related tumors.

## Lipid Regulatory Factor—Insulin/Insulin-Like Growth Factor

Insulin is a protein hormone secreted by islet β cells in the pancreas. Type 2 diabetes is caused by insufficiencies in insulin secretion and insulin resistance. The main therapeutic methods are oral hypoglycemic drugs and insulin injection. However, insulin treatment can cause weight gain at the same time (112), and one of the inducements of type 2 diabetes is obesity, thus, obese type 2 diabetes patients will experience a further increase in obesity after receiving insulin treatment.

Both obesity and insulin resistance can weaken the insulin utilization rate and lead to an increase in blood glucose levels. To reduce blood sugar, β cells of the islets of Langerhans secrete more insulin, which results in hyperinsulinism. Therefore, most obese patients with type 2 diabetes have hyperinsulinism, and higher insulin levels will reduce the level of IGFBP-1 (113), which makes IGF dissociate from IGFBP-1, and then act on IGF-R. Therefore, hyperinsulinism may enhance the bioavailability of insulin-like growth factor (IGF).

Insulin-like growth factor (IGF) was named because of its similar structure to insulin and is a polypeptide with a growth-promoting effect. There are two IGF types in the family: IGF-I and IGF-II. Although the liver is the main site of IGF-I and IGF-II producing, their expression can often be observed in tumor tissues (114), affecting the progression of tumor. Research on insulin-like growth factor and tumor formation and progression occurred earlier than that on insulin. As early as 1984, Myal Y, et al. found that insulin-like growth factor participates in the growth regulation of human breast cancer cells and promotes the growth of cancer cells by binding with corresponding receptors in breast cancer (115). Later studies also found that IGF has a positive role in promoting the growth of tumors and is closely related to the occurrence and development of tumors.

There are two types of receptors for insulin-like growth factors(IGFs): IGF-I receptor (IGF-IR) and IGF-II receptor (IGF-IIR). The insulin receptor is also divided into two subtypes: A (IR-A) and B (IR-B). The physiological role of IR subtypes seems to be determined by their different binding affinities for insulin-like growth factor (IGF), especially for IGF-II (116). The differential expression of insulin receptor and insulin-like growth factor receptor subtypes may help to explain the diversity of signals and functions of insulin and insulin-like growth factor in different tissues and organs. Coincidentally, both IGF-IR and insulin receptor (IR) belong to the tyrosine kinase receptor family (117), with structural and sequence homology, which provides preliminary evidence for the homology between insulin and insulin-like growth factor-1 receptor and the characteristics of oncogenes (114, 115).

### Insulin, Insulin Receptor, and Tumors

**Figure 3:**
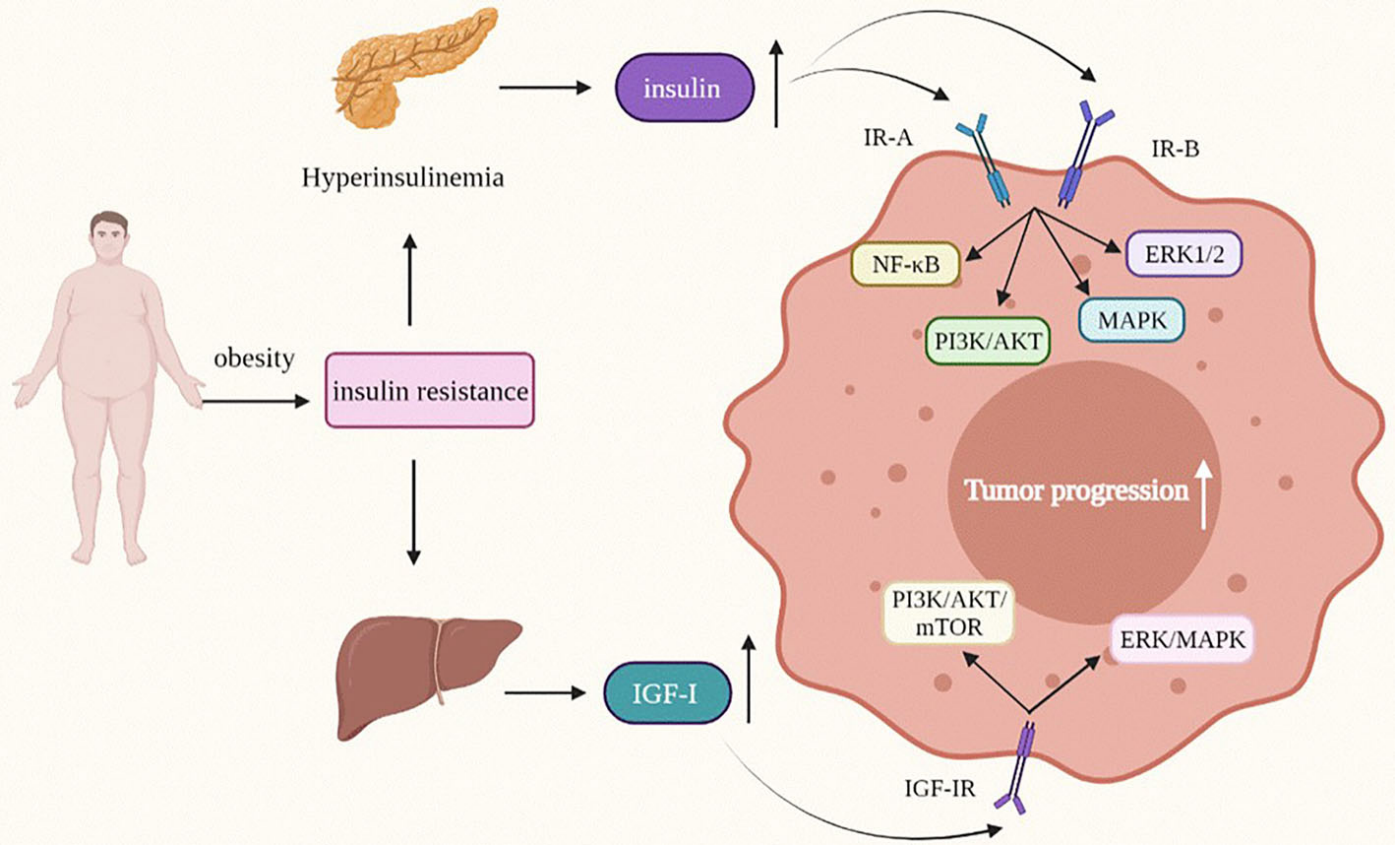
Insulin and IGF-I modify the behavior of obesity-related tumor cells. IGF-I, Insulin-like growth factor-I; MAPK/ERK, mitogen-activated protein kinase/extractor signal regulated kinase; PI3K/AKT, phosphoinositide-3 kinase/protein kinase B.

As a risk factor for the occurrence and development of a variety of cancers, insulin participates in metabolic, cell survival and proliferation signaling pathways (**Figure 3**) (118). Most of its potential mechanisms are to promote cell mitosis, growth and proliferation and inhibit cell apoptosis (119, 120). Insulin receptor (IR) is a key regulatory factor in development, growth and metabolism (121). IR is divided into two subtypes: A (IR-A) and B (IR-B). IR-A is mainly expressed in foetal tissues and the brain, while IR-B expression is the highest in the liver (122). IR-A is often highly expressed in various malignant tumors, including breast cancer (118). The high insulin level in obesity and type 2 diabetes is related to the occurrence and development of various tumors. The hyperinsulinemia/insulin signaling pathway may play a role in the development of breast cancer. An increased fasting insulin level will increase the risk of breast cancer (123), and a higher insulin level can promote breast cancer growth and metastasis through the PI3K/AKT signaling pathway, and promote tumor progression (118). In breast cancer (except for breast fibroadenoma), the expression level of insulin receptor increases, which is one of the independent risk factors for a worse prognosis for breast cancer patients with type 2 diabetes after insulin treatment (124). Although we know that hyperinsulinemia induced by obesity can lead to the occurrence of type 2 diabetes after insulin resistance, which is closely related to the occurrence, development and prognosis of breast cancer, the mechanism of crosstalk among type 2 diabetes, insulin and breast cancer is still unclear, and further research is needed.

The effect of high insulin levels on tumors is reflected not only in breast cancer, but is also a risk factor for pancreatic cancer. A sharp rise in insulin levels due to obesity and type 2 diabetes indicates an increase in the risk of cancer, especially newly developed diabetes, which seems to be closely related to pancreatic cancer (125). In recent years, insulin has been found to affect the progression of pancreatic cancer at different stages. In human pancreatic ductal epithelial cells, insulin can upregulate matrix metalloproteinase-2 (MMP-2) by activating the PI3K/AKT and ERK1/2 signaling pathways and promote the invasion and metastasis of K-RAS mutant human pancreatic ductal epithelial cells (126). In addition, insulin can induce an increase in PLK1 (a serine/threonine kinase widely expressed in eukaryotic cells) through the PI3K/AKT and NF-KB pathways, thus enhancing the proliferation activity of pancreatic duct epithelial cells (127).

In addition to promoting the progression of pancreatic cancer, there is a positive correlation between high insulin levels and the progression of colon cancer. High cholesterol ester content in lipid droplets is an important characteristic of tumors, and insulin can upregulate ACAT1 expression, an important enzyme that plays a role in the cholesterol esterification pathway, to promote the proliferation and migration of colon cancer cells (HT-29 cells) (128). In addition, insulin mediates insulin receptors, up-regulates MMP-2 expression, stimulates insulin receptor substrate-1 (IRS-1), and then activates the PI3K/AKT and MAPK signaling pathways to promote cancer cell proliferation and enhance cancer cell migration ability (129). High insulin levels will not only accelerate the progression of colorectal cancer but also affect its treatment and prognosis. Moreover, clinical research has found that the risk of recurrence and death will increase in patients with stage III colon cancer after consumption of a high-insulin diet, which indicates that cultivation of healthy dietary habits after colorectal cancer surgery is very important, and a diet rich in carbohydrates, protein and lipids that can stimulate insulin secretion should be avoided. Control of insulin levels is helpful for the treatment and prognosis of tumors (130).

### Insulin-Like Growth Factor and Tumors

IGF can be divided into two subtypes: IGF-I and IGF-II. At high insulin levels, the bioavailability of IGF will increase, and IGF can also independently affect the occurrence and development of tumors (**Figure 3**) (131). In the process of tumorigenesis and tumor development, the most important functions of the IGF family include promoting cell proliferation, inhibiting cell apoptosis, regulating cell differentiation and inducing epithelial mesenchymal transition (EMT) phenotype (132–136).

#### Insulin-Like Growth Factor-I (IGF-I) and Tumors

In the control of growth hormone (GH), the liver is the most important place to produce circulating IGF-I (116). As early as 1987, Pollak Mn et al. found IGF-IR overexpression in breast cancer and colon cancer (137). Since then, the relationship between IGF-I and cancer has become the focus of many researchers. The IGF-I/IGF-IR signaling axis can mediate tumor progression through the PI3K/AKT, rapamycin (mTOR), and ERK/MAPK pathways, among others (133–135, 138–140). In triple-negative breast cancer, the IGF-I/IGF-IR signaling axis activates focal adhesion kinase (FAK), which is a nonreceptor cytoplasmic protein tyrosine kinase, and then regulates the expression of the transcription cofactor Yap to promote the growth of triple-negative breast cancer (TNBC) cells (141). IGF-I can induce the EMT phenotype of breast cancer, thus promoting the migration, invasion and metastasis of epithelial tumors. The overall mechanism is very complex and is mainly divided into mechanisms involving the PI3K/AKT pathways and mechanisms involving the RAS/MEK/ERK (134).

The IGF-I/IGF-IR signaling axis not only affects the development of breast cancer, but also significantly affects pancreatic cancer, ovarian cancer, and colon cancer. In pancreatic cancer, IGF-I induces K394 deacetylation *via* the PI3K/AKT/mTOR signaling pathway and stimulates enolase 2 (ENO2) activity by increasing the phosphorylation of histone deacetylase 3 (HDAC3) of S424, a key glycolytic enzyme in glycolysis metabolism, thus promoting liver metastasis of pancreatic ductal adenocarcinoma (PDAC) (138). Although little is known about the relationship between IGF-I and ovarian cancer, we can be sure that IGF-I is involved in the development of epithelial ovarian cancer (EOC). It is worth noting that the influence of IGF-I in EOC is two-sided. On the one hand, IGF-I can increase the expression of snail and slug by activating the PI3K/AKT/mTOR signaling pathway, and snail and slug can induce the downregulation of E-cadherin by interacting with the E-box binding site in the E-cadherin promoter and enhance the proliferation of ovarian cancer cells (139). On the other hand, elevated serum IGF-1 levels have a positive effect on the overall survival rate of EOC patients (140).

Clinical studies have found that the expression of IGF-I and IGF-IR mRNA in colorectal cancer is significantly higher than that in colorectal adenoma and normal tissues (142), indicating that obesity-related colorectal cancer may also be affected by IGF-I. IGF-I can promote cell proliferation and inhibit apoptosis by activating PI3K/AKT and the typical Wnt signaling pathway (135).

At present, many studies on IGF-I/IGF-IR have focused more on the PI3K/AKT signaling pathway than the MAPK/ERK pathway, thus, the signaling relationship between the IGF-I/IGF-IR axis and the MAPK/ERK pathway is still unclear and needs further research and exploration. However, it is clear that overexpression of IGF-I and IGF-IR is involved in the occurrence and development of various obesity-related tumors. In-depth study of this will help in finding more effective diagnostic criteria and treatment methods.

#### Insulin-Like Growth Factor-II (IGF-II) and Tumors

Insulin-like growth factor-II is a multifunctional cell proliferation regulatory factor that can affect the proliferation of tumor cells. In breast cancer, the expression level of IGF-II is positively correlated with the expression levels of the anti-apoptotic proteins Bcl-2, Bcl-XL and survivin (136); thus IGF-II can promote the development of tumors by accelerating tumor growth, inhibiting cancer cell apoptosis and promoting tumor cell metastasis. IGF-II can not only affect the activity of breast cancer but can also generate mitotic signals and anti-apoptosis effects by activating IR and IGF-IR, thus promoting the growth of breast cancer cells (143). IGF-II can promote the proliferation of breast cancer cells in many ways. IGF-II is highly expressed in all triple-negative breast cancer (TNBC) cell lines. It promotes the growth of triple-negative breast cancer cells through autocrine and/or paracrine mechanisms. The mechanism may be related to androgen receptor (AR) and estrogen receptor (ER β) (144, 145). Therefore, two-way targeted therapy for the IGF-II, AR and ER β signaling pathways is expected to be a potential target for TNBC treatment. A difference in IGF-II expression *in vivo* can also lead to different tumor survival rates; however, the relationship between IGF-II and breast cancer prognosis is still unclear (136, 146).

There are several reports on the effect of IGF-II in pancreatic cancer, and most of them are limited to precancerous diagnosis. In previous clinical prospective studies, we observed that there is no correlation between the risk of pancreatic cancer and the level of IGF-II in the plasma before diagnosis (147, 148). However, recent studies have found that there is a negative correlation between a higher IGF-II level and the survival rate of male pancreatic cancer patients before the diagnosis of pancreatic cancer (149), that is, the higher the IGF-II level is, the higher the survival rate of male pancreatic cancer patients. However, the underlying mechanism is not clear.

The IGF-II gene was the first endogenous imprinting gene to be discovered, and the loss of imprinting of (LOI) IGF-II is an early event in colorectal cancer (150, 151). LoI can lead to IGF-II overexpression and activate IGF-IR and AKT1 (151), leading to cancer cell proliferation and promoting progression of colorectal cancer, and overexpression of IGF-II is the main oncogenic factor in colorectal cancer (152). Therefore, the IGF-II–LoI system can be used as a research focus in colorectal cancer, and provide a new idea for targeted treatment of colorectal cancer.

## Crosstalk Between Adiponectin and Leptin, Insulin, and Estrogen

### Interaction of Adiponectin and Leptin in Tumor

Both adiponectin and leptin are secreted by adipose tissue, but unlike leptin, whose expression level is proportional to the total fat content, the expression level of adiponectin is inversely proportional (153). Therefore, compared with normal weight people, the adiponectin/leptin ratio in obese patients is lower, and the risk of tumors (such as breast cancer, pancreatic cancer, and endometrial cancer) is higher (61, 154, 155).In addition, the study found that the expression level of adiponectin was inversely proportional to that of leptin; that is, when adiponectin expression is increased, the level of leptin expression is decreased accordingly. Furthermore, adiponectin can inhibit the proliferation, migration and invasion of breast cancer cells induced by leptin and has anti-tumor activity (156, 157). *In vitro* experiments have also confirmed that lower adiponectin levels in obese people are a prerequisite for leptin to promote tumor growth and promote tumor progression (158). Therefore, the high level of leptin in obese patients is the main reason for tumor progression and metastasis, and increasing the circulating level of adiponectin in the body can break the tumor-promoting effect of leptin, especially supplementing sufficient adiponectin to obese tumor patients to reverse the series of cancer-promoting effects caused by the high concentration of leptin.

The mechanism by which adiponectin antagonizes leptin in promoting tumor is not clear, but studies have found that adiponectin can inhibit the activation of ERK and AKT in the leptin signaling axis; increase the expression and activity of protein tyrosine phosphatase 1B (PTP1B), a physiological inhibitor of leptin signaling, and block the growth of breast cancer cells induced by leptin (156). Leptin often plays a tumor-promoting effect through the STAT3 signaling pathway and stimulates cell proliferation, migration and invasion. Adiponectin can inhibit leptin-induced activation of JAK2 and STAT3 transcriptional activity in a PTP1B-sensitive manner, thereby inhibiting leptin signal transduction and cancer-promoting behavior (159).The anti-inflammatory effect of adiponectin also interferes with the cancer-promoting effect of leptin. Studies have found that globular adiponectin (gAcrp) can inhibit the activation of leptin inflammatory bodies through heme oxygenase-1 (HO-1) signaling, thus preventing the cancer-promoting effect of leptin (160).

### Interaction of Adiponectin and Insulin in Tumors

Obesity-induced insulin resistance and an increased risk of diabetes are closely related to the downregulation of adiponectin levels. Hepatic insulin resistance is the key to predicting diabetes. In 2001, scientists discovered for the first time that adiponectin can enhance the liver sensitivity to insulin and increase the ability of insulin to inhibit glucose production, leading to a decrease in liver glucose output. In addition, liver glucose output determines the blood sugar level, which leads to a decrease in serum glucose levels (161). At the same time, adiponectin can also reduce the risk of insulin resistance by reducing triglyceride content in muscles and the liver (162). Adiponectin plays an important role in regulating the balance between glucose and lipid metabolism. The physiological function of adiponectin indicates that adiponectin is an important hormone to reduce or even prevent insulin resistance and type 2 diabetes. Due to the expression of adiponectin mRNA in obese patients and the lower expression level in plasma, we can treat insulin resistance by supplementing adiponectin in the body to avoid the development of type 2 diabetes.

### Interaction of Adiponectin and Estrogen in Tumor

Adiponectin can interfere with the growth and proliferation of ER-α- negative MDA-MB-231 breast cancer cells and reduce tumor size. However, in obese breast cancer patients, the low expression level of adiponectin makes it a growth factor that acts on ER-α-positive MCF-7 breast cancer cells and upregulates the expression of cyclin D1 in the cells. Cyclin D1 stimulates cancer cell growth and affects the anti-cancer effect of adiponectin. This phenomenon will not occur at high adiponectin concentrations. This is likely because the interaction between ER-α and liver kinase B1 (LKB1) inhibits AMPK activation, and when adiponectin is sufficient, adiponectin will tend to bind to adiponectin receptors instead of ER-α (163, 164). This indicates that when treating obese breast cancer patients, estrogen receptor-α-positive breast cancer should be given more adiponectin supplements to prevent the series of adverse chain reactions caused by adiponectin deficiency. Adiponectin can downregulate the expression of ER-α through the HO-1 signaling pathway, thereby blocking the activation of ER-α induced by leptin, blocking the formation of leptin inflammasomes, inhibiting the effect of leptin in promoting tumor growth, and suppressing cancer (160).

## Treatment

Overall, the increased secretion of leptin, insulin, and estrogen and the decreased expression of adiponectin caused by obesity have a significant effect on the occurrence and development of obesity-related tumors (breast cancer, pancreatic cancer, ovarian cancer, and colorectal cancer). Therefore, reducing body fat content and lowering body mass index should be the primary task of treatment and should run through the entire treatment. Studies have found that long-term aerobic exercise can not only reduce body fat, but also significantly reduce the levels of leptin and estrogen, and increase insulin sensitivity, which is of great significance to improve the prognosis of tumor, such as regular swimming, jogging and cycling exercise (165–167).This is of great benefit for reducing the incidence of tumors, delaying tumor progression, and improving patient prognosis (**Table 2**).

**Table 2:** Summary of Treatment Methods.

Adipokines/Hormones	Treatment method/drugs	Reference
Leptin	inhibiting or blocking the signaling pathway of leptin	(168)
	inhibiting the expression of leptin receptors	(169)
	treating leptin resistance: (1) strengthening exercise and reducing energy intake; (2) reduce endoplasmic reticulum stress	(170, 171)
Adiponectin	thiazolidinedione antidiabetic drugs: pioglitazone and rosiglitazone	(172)
	tamoxifen	(173)
	adiponectin receptor agonists: AdipoRon	(174, 175)
Estrogen	ERα antagonist: fulvestrant	(94, 176)
	ERβ agonist: liquiritigenin and S-equol	(177–179)
	aromatase inhibitors such as letrozole	(180–182)
	metformin	(183)
Resistin	paclitaxel	(184)
	hormone therapy	(185)
MIF	nanoparticles system loaded with small interfering RNA (siRNA)	(186)
	(1) Small molecule disruption of MIF activity; (2) Indirect destabilization of MIF, such as isoxazole; (3) MIF inhibitor, such as 4-iodo-6-phenylpyrimidine (4-IPP)	(187, 188)
	high concentration of glucocorticoid	(187)
	gemcitabine should be avoided	(47)
MCP-1	thymoquinone (TQ)	(189)
	metformin	(49)
	CCR2 inhibitor (PF-04136309) combined with FOLFIRINOX chemotherapy (oxaliplatin irinotecan, leucovorin and bolus fluorouracil)	(190)
Insulin/IGF	metformin	(191–194)
	pioglitazone	(195, 196)

In addition to increasing aerobic exercise and controlling diet, different treatment measures should be taken to target different adiponectin. According to the role of leptin in promoting cancer in obese patients, we can carry out tumor intervention from the following four aspects: (1) inhibiting or blocking the signaling pathway of leptin: Leptin can induce JAK/STAT, MAPK/ERK and PI3K/AKT signaling pathways by binding to its receptor, and promote the occurrence and development of obesity related tumors. However, blocking these three signaling pathways can effectively reduce the tumor promoting effect of leptin (168). (2) inhibiting the expression of leptin receptors: Leptin receptor is related to a variety of signal pathways that stimulate the proliferation and migration of cancer cells, so inhibiting the expression of leptin receptor is a feasible treatment for leptin related tumors. For example, the short peptide analogs of leptin receptor binding site III, as selective leptin receptor inhibitors, play an important role in the process of antigenic and metastatic tumors (169). (3) treating leptin resistance: Leptin resistance, as the name implies, means that although there is a large amount of leptin expression in the body, the brain cannot receive the feedback from leptin due to the decreased sensitivity of the brain to leptin receptors or the decreased stimulation intensity of leptin to receptors, so the brain (hypothalamus) cannot regulate the leptin balance by suppressing appetite, reducing energy intake and increasing energy output And maintain weight stability. Leptin resistance will lead to further weight gain, and the level of leptin expression will continue to increase, which will increase the risk of multiple obesity related tumors and promote tumor progression. Therefore, the treatment of leptin resistance is very important (170). The methods to treat leptin resistance include: (1) strengthening exercise and reducing energy intake; (2) reduce endoplasmic reticulum stress (171).

Adiponectin can inhibit tumor proliferation, accelerate tumor apoptosis, improve the efficacy of chemotherapy drugs, and inhibit the high leptin level caused by obesity and thus plays an anti-tumor role in both indirect and direct ways (36, 197). Therefore, improving the expression level of adiponectin becomes a key step in the treatment of tumors. The use of thiazolidinedione antidiabetic drugs, such as pioglitazone and rosiglitazone, in the treatment of diabetes will also increase the production of adiponectin in the body, thus inhibiting the proliferation, migration and invasion of breast cancer (172).In addition, as the preferred drug for the treatment of estrogen receptor-positive breast cancer, tamoxifen can not only block the interference of adiponectin by Erα but also increase the expression level of adiponectin and reduce the level of leptin, and thus plays an important role in the treatment of breast cancer (173). Finally, adiponectin receptor agonists can inhibit tumor progression by inhibiting cell growth and promoting apoptosis (174, 175). Recent studies have found that these agonists can induce the production of superoxide anion by activating ripk1 and ERK1/2, leading to rapid mitochondrial dysfunction resulting in pituitary necrosis and thus inhibiting the growth of pancreatic cancer (198).

Before menopause, estrogen secreted by the ovary is closely related to the progression of breast cancer and ovarian cancer, and an imbalance in the expression ratio of ERα and ERβ plays an important role. Thus, inhibition of ERα expression and upregulation of ERβ expression are of great significance in the treatment of hormone related tumors. Therefore, the ERα antagonist fulvestrant and the ERβ agonist liquiritigenin and S-equol have the potential to treat estrogen-related tumors (breast cancer and ovarian cancer) (94, 177, 178). Fulvestrant, selective estrogen receptor downregulator (SERD), is mainly used in the treatment of postmenopausal estrogen receptor positive metastatic breast cancer (176). Clinical studies have found that low dose (25mg/kg) of fluvistrane can achieve the anti-tumor effect, but compared with high dose (200mg/kg), the down-regulation of estrogen receptor is not obvious (176). As natural ERβ agonists, liquiritigenin and S-equol can not only reduce tumor cell migration and invasion, promote cell apoptosis, but also increase the sensitivity of ovarian cancer cells to cisplatin and paclitaxel (178). Therefore, finding the optimal drug concentration for anti-tumor effect and down-regulation of ER is the focus of our future research. Although flavonoid liquiritigenin is not widely used clinically, as a selective estrogen receptor β agonist, it can significantly reduce the invasion and metastasis ability of breast cancer (179). Therefore, appropriate clinical drug experiments can be carried out to observe the therapeutic effects. After menopause, estrogen is mainly secreted by adipose tissue, and inflammatory factors secreted by adipose tissue can promote the expression of aromatase (72). At the same time, aromatase can convert androgen into estrogen. Therefore, to reduce the expression level of estrogen and achieve the purpose of prevention and treatment of postmenopausal tumors, aromatase inhibitors such as letrozole are an indispensable part of treatment (180). Aromatase inhibitors can inhibit the growth of ER tumor cells by acting on aromatase, or even on ER and AR (androgen receptor), thus significantly prolonging the survival time of tumor patients with high ERα expression (181, 182). Therefore, it is a good targeted therapeutic drug. However, while using aromatase to reduce the level of insulin expression, regular monitoring of estrogen levels is required. Patients should be discontinued when the estrogen level is lower than normal, which is because the lack of estrogen will lead to the imbalance of glucose metabolism and lipid metabolism. In severe cases, it will cause or aggravate insulin resistance, lead to abdominal obesity and even metabolic syndrome. However, metformin can avoid these side effects, because on the one hand, metformin can reduce the size of tumor and inhibit the formation of new tumor, on the other hand, it can reduce liver fat accumulation and strengthen metabolism (183). Therefore, in order to better treat tumors, reduce the expression level of estrogen, and reduce the side effects of drug use, we can use aromatase inhibitors in combination with metformin.

It can be seen from the above that resistin is an independent risk factor for tumorigenesis and tumor development. When patients with high serum resistin receive treatment, reducing the level of resistin in the body is a key step, and paclitaxel, the treatment drug for advanced refractory breast cancer, can reduce the level of serum resistin (184). This is of great benefit to improving the prognosis of patients and improving the quality of life of patients. Hormone therapy for breast cancer patients with serum high resistin can reduce the recurrence rate, while radiotherapy and chemotherapy are not effective (99). In addition, doxorubicin should not be used to treat patients with high resistin, because resistin can protect tumor cells from the toxicity of doxorubicin by up regulating the expression and phosphorylation of STAT3 and reverse the apoptosis effect of doxorubicin on tumor cells, which will improve resistance of patients to doxorubicin (185).

Since MIF is a secretory protein, therapy targeting MIF or its receptor may effectively inhibit tumor growth. For the treatment of MIF overexpression tumor, inhibiting MIF is the key measure. A nanoparticles system loaded with small interfering RNA (siRNA) can effectively reduce MIF expression in breast cancer cells and macrophages, which can induce anti-tumor immune response by reducing systemic immunosuppression (186). In tumor treatment research, it is found that there are three main methods to inhibit the biological function of MIF: (1) Small molecule disruption of MIF activity; (2) Indirect destabilization of MIF, such as isoxazole; (3) MIF inhibitor, such as 4-iodo-6-phenylpyrimidine (4-IPP), which can block MIF/CD74 signal axis (187, 188). However, these three treatment methods are still in basic research, lacking a large number of reliable clinical studies as support. Although some studies have found that high concentration of glucocorticoid can down regulate the expression of MIF (187), the current research is not enough to show that high concentration of glucocorticoid is effective in the treatment of high level of MIF, and the optimal concentration of glucocorticoid is not known. Follow up studies can focus on the use of high concentrations of glucocorticoids to inhibit MIF expression in the treatment of tumor. It is worth noting that although gemcitabine is the standard chemotherapy for PDAC, the high expression of MIF can reduce the sensitivity of tumor cells to gemcitabine (47). Therefore, gemcitabine should be avoided in the treatment of tumors with high levels of MIF. At present, the research of anti MIF therapy is far behind the targeted research of other factors, so more drug clinical trials are needed.

Inflammation has been identified as an important factor in the development of solid malignancies. Therefore, MCP-1, which can induce macrophage recruitment, has become a potential target for tumor therapy. Thymoquinone (TQ), the major constituent of Nigella sativa oil extract, can inhibit the proliferation and induce apoptosis of pancreatic ductal adenocarcinoma cells by down regulating MCP-1 (189). TQ provides a new strategy for anti-inflammatory and anti-tumor therapy. In addition, recent study has found that ovarian cancer mice treated with metformin have a certain degree of inhibition of tumor metastasis and prolong the survival time of the mice, and found that the expression level of MCP-1 in the tumor microenvironment is reduced at the same time (49). It is speculated that metformin may inhibit the metastasis of ovarian cancer cells by inhibiting the secretion of MCP-1 from adipocytes. Clinical trials should be conducted to test its therapeutic effect. In most cases, MCP-1 works by binding to CCR2, so when treating tumors with high expression of MCP-1, the treatment of inhibiting CCR2 is essential. The clinical drug experiment found that CCR2 inhibitor (PF-04136309) combined with FOLFIRINOX chemotherapy (oxaliplatin irinotecan, leucovorin and bolus fluorouracil) was safe and reliable, which could reduce the accumulation of tumor related macrophages and change the tumor microenvironment (190). On the whole, for the treatment of tumors with high expression of MCP-1, on the one hand, it can reduce the expression of MCP-1, on the other hand, it can antagonize the CCR2 receptor, so as to inhibit tumor cell invasion and metastasis and reduce the accumulation of macrophages in the tumor microenvironment.

It is well known that most diabetic patients have obesity. Obesity and diabetes promote the occurrence and development of obesity related tumors, therefore, many cancer patients are accompanied with diabetes mellitus. For this kind of patients, the first choice should be drugs that can not only control blood glucose concentration, but also hinder tumor progression, such as metformin and pioglitazone. Metformin, the first-line treatment for diabetes, can prevent weight gain and play an anti-tumor role in a low glucose environment (191). In addition, metformin can slow down the growth of cancer cells by activating AMPK to inhibit mammalian rapamycin target (mTOR), thus reducing the adverse effects of obesity and diabetes on cancer (192). Furthermore, meta-analysis study showed that the mortality of cancer patients with diabetes mellitus was reduced by 34% after using metformin, and the use of metformin in adjuvant treatment of colorectal cancer patients can significantly improve the prognosis (193, 194). Pioglitazone, which is also an antidiabetic drug, plays an important role in reducing insulin resistance in peripheral tissues and the liver. In recent years, pioglitazone has been found to activate peroxisome proliferator activated receptors- γ (PPAR- γ), a tumor suppressor gene, which can also inhibit the proliferation and migration of breast cancer by mediating JAK2/STAT3 pathway (195, 196). Although both metformin and pioglitazone have antitumor effects, there are not enough studies to verify whether the combination of metformin and pioglitazone can increase the efficacy, which needs further study.

The tumor microenvironment is a very complex environment, which indicates that the progression of tumors is not only affected by a single factor. Therefore, the treatment of tumors needs to be considered comprehensively, and drugs cannot be administered separately. From the above treatment summary, it is not difficult to see that metformin has a wide range of applications. It can not only reduce the adverse effects of obesity and diabetes on cancer, but also achieve the purpose of inhibiting tumor progression by reducing the expression of estrogen and MIF. However, there is currently a lack of more in-depth studies on the specific mechanism of metformin in the treatment of tumors, and there is also a lack of systematic clinical studies on the changes in the prognosis and survival of patients treated with metformin, so more clinical trials need to be implemented.

## Conclusions

Obesity is an independent risk factor for tumor occurrence and development. In today’s society, as the number of obese people increases, it is important to understand the mechanism of obesity that affects tumor occurrence and development. At present, we have found that obesity not only up-regulates the expression level of adipokines that promote cancer, but also down-regulates the tumor suppressor adiponectin to stimulate tumor progression. On the basis of insulin resistance, obesity can increase the expression level of insulin and IGF, and promote the proliferation, invasion and metastasis of tumor cells. Therefore, with the deepening of our understanding of the mechanism of obesity related tumor, it is urgent to find a suitable, appropriate and effective treatment. However, the current research is still incomplete, we need to further explore the mechanism of tumorigenesis and development of tumors and implement more extensive clinical drug experimental research.

## Author Contributions

XP wrote this manuscript and DC revised the manuscript. All authors contributed to the article and approved the submitted version.

## Funding

The National Natural Science Foundation of China (grant no. 81572956), The Jiangsu Provincial Science and Technology Supporting Program (grant no. BE2017096).

## Conflict of Interest

The authors declare that the research was conducted in the absence of any commercial or financial relationships that could be construed as a potential conflict of interest.

## Publisher’s Note

All claims expressed in this article are solely those of the authors and do not necessarily represent those of their affiliated organizations, or those of the publisher, the editors and the reviewers. Any product that may be evaluated in this article, or claim that may be made by its manufacturer, is not guaranteed or endorsed by the publisher.
